# Long-term dynamic topographic support during post-orogenic crustal thinning revealed by stable isotope (δ^18^O) paleo-altimetry in eastern Pyrenees

**DOI:** 10.1038/s41598-020-58903-w

**Published:** 2020-02-10

**Authors:** Damien Huyghe, Frédéric Mouthereau, Loïc Ségalen, Marc Furió

**Affiliations:** 1 Géosciences Environnement Toulouse, CNRS, IRD, Université Paul Sabatier Toulouse 3, 14 Avenue Edouard Belin, 31400 Toulouse, France; 2 MINES ParisTech, PSL University, Centre de Géosciences, 35 rue St Honoré, 77305 Fontainebleau, Cedex France; 3 Sorbonne Université, CNRS-INSU, Institut des Sciences de la Terre Paris, ISTeP, F-75005 Paris, France; 4 Institut Català de Paleontologia Miquel Crusafont, Universitat Autònoma de Barcelona, C/de les Columnes s/n, 08193 Cerdanyola del Vallès, Barcelona Spain; 5 Departament de Geologia, Universitat Autònoma de Barcelona, 08193 Cerdanyola del Vallès, Barcelona Spain

**Keywords:** Geochemistry, Structural geology, Tectonics

## Abstract

Understanding the geodynamic and Earth surface processes at the origin of post-collisional surface uplift in mountain ranges requires reconstruction of paleo-elevation. Here, we focus on the topographic evolution of the Cerdanya Basin in the eastern Pyrenees formed by post-orogenic extension during the Late Miocene. Stable isotope (δ^18^O) analyses of small rodent teeth and biogenic carbonates show the basin uplifted by 500 m since 6.5 Ma. These new paleoaltitudes constraints when combined with the regional geology and geophysical data reveal the anomalously high topography of the region is the result of density changes in the sublithospheric mantle associated with crustal thinning and then opening of Gulf of Lion during the Chattian-early Burdigalian.

## Introduction

The drivers of post-collisional topographic uplift of mountain ranges, when plate convergence has ceased, are debated. Main processes invoked include the thinning of the dense lithosphere by sublithospheric deblobbing, delamination of a sinking slab, and replacement by the lighter asthenosphere^1^ or isostatic rebound caused by enhanced erosion^2^. Where changes in plate kinematics from contraction to extension occur, post-orogenic crustal thinning should promote subsidence not uplift. The case of the eastern Pyrenean mountain belt is particularly relevant because the region recorded crustal thinning during the opening of the Mediterranean Sea (Gulf of Lion) and currently shows high topography in presence of an attenuated crustal root. This is reflected by the isostatic anomalies that reveal a non-isostatic dynamic support of the topography^3^. Mechanical removal of the mantle lithosphere has been proposed^4,5^, but details on the timing and amount of uplift are lacking to further discuss the drivers of post-orogenic surface uplift.

Collision in the Pyrenees occurred from Late Cretaceous to the Early Miocene^6–8^. Low-temperature thermochronological constraints from the Central Pyrenees define that exhumation, possibly enhanced by climatic changes at the Eocene-Oligocene transition^9^, accelerated at 37–30 Ma (>2.5 km/Myr)^10,11^. Paleo-elevation of the Pyrenees is estimated to 2 ± 0.5 km in the Lutetian^12^. This value is in agreement with other estimates of maximum 2 km in the Middle Lutetian based on flexure modelling^13^, although a more recent flexural study considers that this altitude might have been reached later in the Late Eocene^14^.

**Figure 1:**
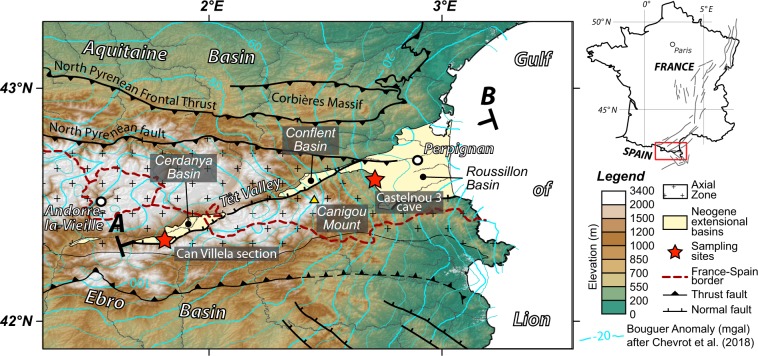
Topography and structural map of the eastern Pyrenees showing the location of the Neogene Cerdanya, Conflent and Roussillon extensional basins. The location of sampling sites where mammal teeth, charophyte oogonia and gastropod shells have been sampled is reported. Isolines of the Bouguer anomaly are from^3^. The west European Cenozoic rift system is reported in the map inset after^43^.

Since the Chattian-Aquitanian, back-arc extension related to  slab retreat led to the opening of the Gulf of Lion^15^ and affected the eastern prolongation of the Pyrenees. From that period onwards, the eastern Pyrenees recorded a different tectonic evolution in comparison with the central Pyrenees. An uplift of about 1 km has been inferred from palynological constraints but its initiation at 10 Ma (Tortonian) or ca. 6 Ma (Messinian) is not resolved^16^. Extension in the eastern Pyrenees is documented by the 22 km-thick crust in the Roussillon Basin, east of the Têt Fault^3^. Despite half of the crust has been removed during extension, the topography stands well above sea level at 2 km on average (e.g. Canigou massif; Fig. 1), indicating a component of the topography is dynamically supported. This is further indicated by the large negative Bouguer anomaly of about −100 mGal (Fig. 1) that led^3,17^ to propose the removal of the dense lower crust or/and ascent of an abnormally buoyant and hot lithospheric mantle. For comparison, the rest of the Pyrenees exhibits a homogenous mean elevation with the highest peaks above 3 km that are isostatically compensated by a 44 km crustal root^3^.

The central and eastern Pyrenees are further characterized by the presence of late Oligocene-early Miocene highly-elevated low relief surfaces, considered remnants of a single composite planation surface recently dissected^18^. There is a great debate about how these surfaces were created and especially at which elevation. Two main hypotheses are proposed. The first one assumes a formation at low elevation and an uplift of the eastern Pyrenees since the early Miocene^5,19^. In this interpretation, mantle thinning is thought to have caused uplift of summit peneplain from low-elevation near sea-level^20^ or at ca. 750 m to present-day 2.4–2.9 km since 12 Ma^5^. The second hypothesis postulates a control by the piedmont sedimentation and the development of a planation surface at high elevation, implying only a limited surface uplift of 400 m in response to post-orogenic erosional rebound^21,22^. Note that because late Miocene normal faulting accommodated little extension, it is considered to have played a subordinate role.

In the present study, we aim to provide new calibration of the post-orogenic paleo-elevation evolution of the Pyrenees. To this aim, we compare the stable isotope composition of material mineralized from meteoric water at different elevations^23,24^. We targeted the Roussillon Basin that remained at low elevation since the Miocene and the Cerdanya basin which current elevation ranges between 1000 and 1200 m (Fig. 1) and that are both characterized by contemporaneous Neogene sedimentary filling. Given the lack of material classically used for paleo-elevation reconstruction like soil carbonates or authigenic minerals, we developed an unconventional approach based on δ^18^O measurements of mammal teeth^25^ and on freshwater algae (charophytes oogonia) and terrestrial gastropod shells.

## Results and Discussion

### Stable isotopic constraints on rodent teeth

**Figure 2:**
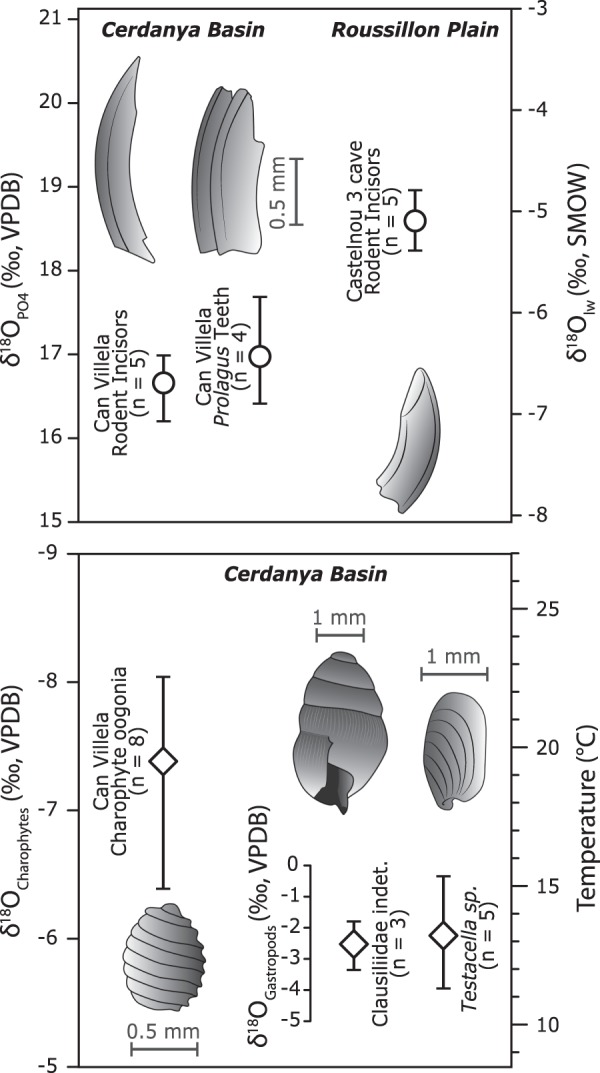
Oxygen stable isotopes results (δ^18^O_PO4_) for the mammal teeth of the Cerdanya basin and Roussillon plain converted in δ^18^O of the local water (δ^18^O_lw_). The δ^18^O of the charophytes and the gastropods converted to temperatures are reported.

Rodent incisors (n = 5) and lagomorph teeth (n = 4) from the Can Vilella section (Cerdanya Basin) yielded mean δ^18^O_PO4_ composition of 16.6 ± 0.3‰ and 17 ± 0.5‰ (Fig. 2). For the Castelnou 3 cave (n = 5) (Roussillon Basin), a mean δ^18^O_PO4_ value of 18.6 ± 0.3‰ were obtained. The charophyte oogonia (n = 8) yielded mean δ^18^O_ch_ of −7.4 ± 0.6‰. For the pulmonate gastropods, the clausilid shells (n = 3) and the *Testacella* specimens (n = 5) have mean δ^18^O_Ga_ of −2.5 ± 0.5‰ and −2.2 ± 1.4‰ respectively (Fig. 2). To convert the δ^18^O_PO4_ of teeth to δ^18^O of the local water (δ^18^O_lw_), we adopt the Eq. (1) of^26^, established from the analysis of west European living small rodents:

1 $${{\rm{\delta }}}^{18}{{\rm{O}}}_{{\rm{PO}}4}=1.21(\pm 0.2){{\rm{\delta }}}^{18}{{\rm{O}}}_{{\rm{lw}}}+24.76(\pm 2.7)$$

where δ^18^O_PO4_ is the δ^18^O value of the phosphate of the rodent teeth and δ^18^O_lw_ is the δ^18^O isotopic composition of the local water. We deduce from Eq. (1) δ^18^O_lw_ values of −6.6 ± 0.3‰ and −5.1 ± 0.2‰ for Can Villela and Castelnou 3 sections, respectively (Fig. 2).

The Eq. (2) of^27^ allows estimating summer temperature values for lake waters from the charophytes based on the δ^18^O_lw_ obtained on mammal teeth:

2 $${\rm{T}}(^\circ {\rm{C}})=15.7-4.36({{\rm{\delta }}}^{18}{{\rm{O}}}_{{\rm{ch}}}-{{\rm{\delta }}}^{18}{{\rm{O}}}_{{\rm{lw}}})+0.12{({{\rm{\delta }}}^{18}{{\rm{O}}}_{{\rm{ch}}}-{{\rm{\delta }}}^{18}{{\rm{O}}}_{{\rm{lw}}})}^{2}$$

where δ^18^O_ch_ is the isotopic composition of the charophytes. We infer a mean summer temperature of 19.5 ± 2.6 °C. Mean annual air soil temperature is calculated based on δ^18^O of the terrestrial gastropods (δ^18^O_Ga_) according to^28^:

3 $${\rm{T}}(^\circ {\rm{C}})=1.15{{\rm{\delta }}}^{18}{{\rm{O}}}_{{\rm{Ga}}}+15.79$$

It yields mean annual air temperature of 12.9 ± 0.6 °C and 13.6 ± 1.6 °C for the clausilids and *Testacella*, respectively. This is slightly lower than the 15.5 to 19.8 °C obtained for the MAT from pollen analyses^16^.

Climate models have shown that modern atmospheric circulations in western Europe, characterized by dominant moisture source from the north Atlantic, were established during the late Miocene^29^. Thus, the modern isotope lapse rate established for the eastern Pyrenees^30^ is used to extrapolate the δ^18^O_lw_ values to estimate a ∆-elevation paleogradient between the two sites during the late Miocene. Measurements from modern small rivers yielded a gradient of −3.76‰/km for the δ^18^O^30^. We infer a mean ∆δ^18^O_lw_ of −1.5‰ for the Miocene samples that corresponds to an altitude difference of ∆H = 396 ± 50 m according to the modern isotope lapse rate.

**Figure 3:**
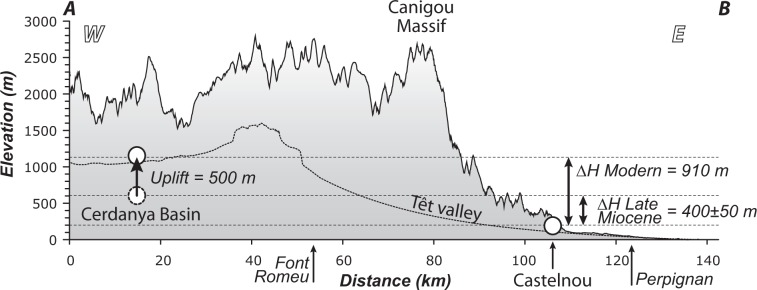
Topographic profile of the eastern Pyrenees along the Cerdanya Basin and Têt Valley and along the highest peaks of the southern flank of the Têt Valley (see location in Fig. 1). This profile presents the modern elevation of the two sampling sites and the elevation of the Cerdanya Basin during the Late Miocene (6.5 Ma) deduced from the δ^18^O composition of mammal teeth.

The modern difference of elevation is 910 m between the two sites (Fig. 3). This result therefore suggests that the Cerdanya Basin uplifted by about 500 m since 6.5 Ma. The basin is currently at 1100 m, we thus estimate a paleo-elevation of 600 m consistent with altitude inferred from pollen floras^16^. The concordant results obtained based on two independent approaches emphasize the robustness and the accuracy of the calculated paleo-elevation. We derive a surface uplift rate of 0.07 mm/a since 6.5 Ma in the range of 0.06–0.12 mm/yr obtained by^16^ for the same basin and close to uplift rates of 0.08–0.19 mm/yr obtained for the Central Pyrenees^31^. Such a rate is also close to incision rates of 0.05–0.09 mm/yr since 5 Ma obtained in the Têt river canyon (current elevation ~400 m)^32^. Pollen data further suggested the relief between the Cerdanya Basin and the surrounding high mountains has not changed since 10 Ma^16^. This inference is supported by the good preservation of the late Miocene sediment infill of the Cerdanya Basin at high elevation and the low rate of erosion on the flank of the mountain range. With a maximum altitude of the region close to 2–2.5 km during the Messinian, the eastern Pyrenees were virtually in the same isostatic state as today, that is the topography was not isostatically compensated by a crustal root. This argues that the major geodynamic changes at the origin of post-orogenic uplift must have started before 10 Ma.

### Post-orogenic evolution of the pyrenees and the opening of the gulf of Lion

Time-temperature paths reconstructed from the eastern Pyrenees show that crystalline Paleozoic basements on both sides of the Têt fault exhumed before the Burdigalian (18 Ma) with up to 2 km of exhumation during late Oligocene (26–27 Ma^33^), likely related to normal faulting with a component of left-lateral strike-slip movement^15^.The Conflent Basin, along the northern segment of the Têt Fault (Fig. 1) preserves remnant of a thick assemblage (~1 km) of coarse clastic sediments^32^: the Marquixanes Formation of Aquitanian age sourced from the surrounding Variscan massifs and topped by the Lentilla alluvial series dated to the early Burdigalian based on mammal fauna^34^. The Têt Fault therefore exhumed the Variscan basement and thinned the crust prior to the Burdigalian, like other N70°E-striking faults recognized offshore.

**Figure 4:**
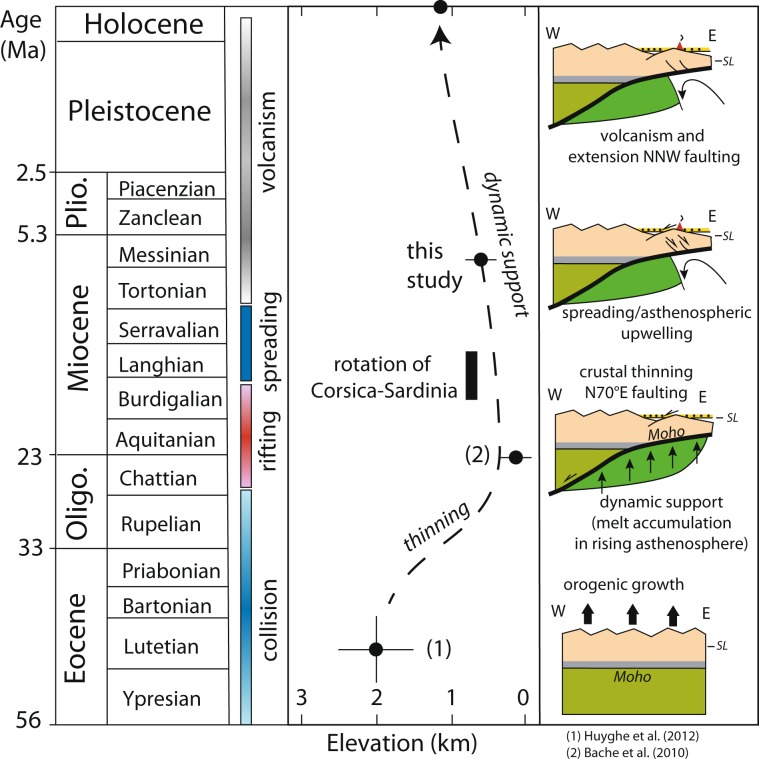
Temporal chart of the main tectonic events in the eastern Pyrenees plotted against the elevation history inferred from this study and other tectonic-stratigraphic constraints presented in the text. Sketches illustrate across a W-E-oriented transect the deep crustal and mantle processes at the origin of the topography of the eastern Pyrenees.

This rifting phase ended in the Burdigalian as indicated by a regional erosional surface recognized in the Gulf of Lion onto which the transgressive shallow-marine post-rift Burdigalian series were deposited^35^. This places an additional elevation constraint near sea-level in the Chattian-Early Burdigalian (Fig. 4). The mapping of the Burdigalian erosional surface offshore of the Gulf of Lion^35,36^ reveals subaerial erosion occurred on a crust that was moderately to extremely thinned in the SE direction (30 < h_c_ < 5 km; stretching factor 1.4 < β < 9). The elevation of the Gulf of Lion rifted margin was therefore anomalously high and flat in the late Oligocene-Early Burdigalian. In the eastern Pyrenees, the present-day crustal thickness below the Têt Fault ranges between 30 and 40 km. It is thicker below the Cerdanya Basin and thinner below the Conflent Basin, and is only 22 km in the Roussillon Basin. Geophysical data therefore indicate that crustal thinning in the eastern Pyrenees and in the Gulf of Lion did not lead to the subsidence predicted by the McKenzie’s model^37^, otherwise the whole region would have been buried several km below sea-level. Pre-break-up surface uplift that does not fit the subsidence effect of thinning the crust (McKenzie stretching model) is documented on many rifted margins. This requires processes leading to density reduction like serpentinization of the exhumed mantle, mantle phase transitions to lighter mineral phases and the trapping of melt in the rising asthenosphere before breakup are required^38^. We infer that similar processes did occur in the eastern Pyrenees and the Gulf of Lion in order to keep the region close to sea level.

Following the early Burdigalian, however, the Gulf of Lion recorded a rapid post-rift subsidence coeval with oceanic spreading in the Ligurian-Provençal Basin and rotation of Sardinia occurred between 20.5 and 15 Ma^39^. The paleo-elevation constraints obtained in this work show that after the onset of oceanic spreading in the Gulf of Lion, the eastern Pyrenees continued to be uplifted. Differential vertical movements between the Gulf of Lion and the eastern Pyrenees likely triggered post-rift normal faulting that led to the development of the Cerdanya Basin during the Tortonian (12–9 Ma). The Late Miocene reactivation of the Têt Fault as a right-lateral strike-slip fault^40^ was contemporaneous with the deposition of 400–800 m of non-marine sediments in the Cerdanya Basin^41^. In the Roussillon Basin, a maximum of 800–900 m of post-Messinian sediments is preserved^42^. The formation of the Cerdanya Basin was synchronous with normal faulting along the oblique NNW-trending Transverse Fault system in the Sierras Transversales^43^, volcanism in Emporda (10–9 Ma) and Selva (7–2 Ma) region, North-East Catalonia. Magmatism continued with the Olot (Garrotxa) volcanic system (0.7–0.11 Ma), an intraplate alkaline basaltic volcanism with close affinities to the volcanic system of the French Massif Central and Calatrava, Central Spain^44^.

Because late Miocene normal faulting occurred when the Cerdanya Basin was at elevation, the Tortonian-Messinian extension appears to be a consequence rather than a cause of the regional uplift. The post-Messinian uplift of 500 m resolved from this study therefore represents a fraction of the long-term regional uplift that initiated in the Aquitanian-Late Burdigalian (20 Ma) when the eastern Pyrenees were close to sea-level (Fig. 4). This result reveals that the short-lived (5 Myr) initial back-arc rifting event was the main driver of the dynamic support of the topography. Because the region was close to sea-level in the late Oligocene-early Miocene then uplifted in the Late Miocene, a post-orogenic piedmont sedimentation could hardly be maintained, thus precluding the preservation of pre-Late Miocene planation surface. Other factors such as flexural uplift in the footwall of the Transverse Fault system or erosional unloading during the Late Miocene may have played a role, but altogether are not the drivers of the topographic evolution of the eastern Pyrenees.

## Conclusion

Paleo-elevation constraints resolved from stable isotopic analyses indicates that the Cerdanya Basin, one of the main valleys of eastern Pyrenees, was at 600 m above sea level during the Messinian, 500 m below its current elevation. Because most of the relief was established at this time, we argue for a moderate late Miocene uplift of the summit planation surface of 500 m. Tectonic-stratigraphic relationships further indicate the pre-6.5 Ma topography was built on an older landscape inherited from the Chattian-early Burdigalian rifting episode that gave birth to opening of the Gulf of Lion. The non-isostatic processes required to support the current topography are therefore the consequence of a short-lived but major geodynamic event at the origin of both crustal thinning and density changes in the mantle. These new paleo-elevation constraints together with other geological data in the region suggest the uplift was a long-term process initiated in the Late Burdigalian in response to pre-breakup uplift in the Gulf of Lion. Following oceanic spreading of the Gulf of Lion and the rotation of Sardinia, the Tortonian extension associated with transcurrent deformation and volcanism was responsible for the last stage of topographic growth of the eastern Pyrenees.

## Method

### Paleoaltitude and paleotemperature reconstructions

The method relies on the comparison of the stable isotope signature of Late Miocene mammal teeth preserved in two basins at the eastern termination of the Pyrenean range: the Roussillon Basin that remained at low elevation since the Miocene and the Cerdanya basin which current elevation ranges between 1000 and 1200 m. The basic principle of isotopic paleoelevation reconstruction lies on the direct dependency of the δ^18^O and δD of rain with elevation, following the Rayleigh distillation behavior^45^. Paleoelevation can thus be quantified from the analysis of mineralization that precipitate from meteoric waters^46^. A classical approach consists of analyzing nodule soils or roots carbonates in sedimentary basins, clay minerals from fault zones or authigenic minerals mineralized at different elevation^24^. However, such material is rarely preserved syn-orogenic deposits and paleoelevation are thus often difficult to reconstruct. In this work, we adopt an approach combining mammal remains (rodent and lagomorph teeth) with biogenic carbonates. Small mammals are homoeothermic animals living in small areas so the δ^18^O of their biominerals reflects both the life-long δ^18^O composition of their body water^47^ and the surface water of their living area δ^18^O_w_^25^. From the local δ^18^O_w_, the δ^18^O analyses of non-homoeothermic biogenic carbonates allow constraining paleotemperatures^48^. One of main challenge when reconstructing paleoelevation is to carefully take into account climatic parameters changes through time^49^. To minimize this impact we have compared the δ^18^O of rodent teeth of two sites, one that remained at low elevation and one that was potentially uplifted. We derive a paleo-∆δ^18^O_w_ that could be converted to ∆-elevation^23^. Geochemical results are provided in Supplementary Dataset 1. Fossils were sampled from two contemporaneous deposits located in the eastern Pyrenees. First, we analyzed rodent incisors of the Castelnou 3 cave (n = 5), located in the Roussillon Plain at low elevation (~200 m), which preserved sediments deposited near the shoreline and attributed to the late Miocene (~6.5 Ma) by biostratigraphic approach^50^ (Fig. 1). We also sampled fossils from late Miocene alluvial to lacustrine deposits of the Can Villela section of the Cerdanya extensional Basin^41^ (Fig. 1). These deposits have been attributed by magnetostratigraphy and biostratigraphy to Chron C3An.2n or C3An.2n, i.e. 6.5 or 6.1 Ma^41^. Teeth from the species *Prolagus michauxi* (Lagomorpha) (n = 4) and undetermined rodent incisors (n = 5) were analyzed (Fig. 2). This outcrop also yielded charophyte oogonia (freshwater green algae) of the species *Lychnothamnus barbatus* (n = 8). Oogonia are the female reproductive organs and they are preserved as small calcitic spheres biomineralized in small lakes or ponds during the warmer weeks. Their δ^18^O allow constraining summer freshwater temperatures^51,52^. We also analyzed gastropods from the family Clausilidae (n = 3), which are small terrestrial gastropods frequently observed around the Mediterranean Sea. We also obtained land snails from the genus *Testacella* (n = 5), corresponding to small slugs living in soils with a reduced shell located at the posterior end of their bodies. Description of the sampling sites and photographs of the samples analyzed in this work are provided in Supplementary Dataset 2. 

Uncertainties are provided both for paleoelevation and paleotemperature estimations. For paleoelevation values, we took into account the standard deviation of the δ^18^O_PO4_ values obtained from the analysis of the rodent teeth and the uncertainty related to the modern isotope lapse rate^30^. Concerning the paleotemperature values, we consider the standard deviation of the mean of all δ^18^O values for a given species.

### Geochemical analyses

Mammal teeth stable isotope analyses were performed at the Biogéosciences Laboratory of the University of Burgundy (Dijon, France). The teeth were ultrasonically cleaned and residual sediment was removed with a Dremel^©^ tool. The teeth were crushed into powder in an agate mortar and pestle, and aliquots of powdered apatite (1 mg) were dissolved in nitric acid and chemically converted to Ag_3_PO_4_ using the method described by^53^. Oxygen isotope ratios were measured on CO using a High Temperature Pyrolysis Analyzer (Elementar Pyrocube) connected online to an Elementar Isoprime mass spectrometer. All δ^18^O values are reported in per mil relative to V-SMOW (Vienna Standard Mean Ocean Water) by attributing a value of 21.7‰ to NBS120c^54^. Accuracy and reproducibility (≤±0.3‰, 2 σ) were monitored by multiple analyses of Ag_3_PO_4_ from NBS120c.

Charophyte oogonia and gastropods were measured at the Institut des Science de la Terre de Paris (ISTeP, Sorbonne University, Paris, France). Each oogonium was observed and crushed under a binocular glass to prevent any recrystallization or sedimentary filling. Gastropod shell preservation was tested by X-Ray diffraction. Each individual carbonate powder sample (80 µg) was reacted with a 100% anhydric orthophosphoric acid at 70 °C in a Kiel IV carbonate device. Stable isotope analyses were performed on a DELTA V mass spectrometer. Isotope values are reported in conventional delta (δ) notation relative to the Vienna Peedee Belemnite (VPSB) standard. We used an internal standard (marble) calibrated to the international standard NBS-19. Precision is ±0.1‰ for δ^18^O. Geochemical results are provided in Supplementary Dataset 1. 

## Supplementary information

Supplementary Dataset 1.

Supplementary Dataset 2.

## References

[1] Platt JP, England PC (1994). Convective removal of lithosphere beneath mountain belts; thermal and mechanical consequences. Am. J. Sci..

[2] Champagnac JD, Molnar P, Anderson RS, Sue C, Delacou B (2007). Quaternary erosion-induced isostatic rebound in the western Alps. Geology.

[3] Chevrot S (2018). The non-cylindrical crustal architecture of the Pyrenees. Sci. Rep..

[4] Lewis CJ, Vergés J, Marzo M (2000). High mountains in a zone of extended crust: Insights into the Neogene-Quaternary topographic development of northeastern Iberia. Tectonics.

[5] Gunnell Y, Zeyen H, Calvet M (2008). Geophysical evidence of a missing lithospheric root beneath the Eastern Pyrenees: Consequences for post-orogenic uplift and associated geomorphic signatures. Earth Planet. Sci. Lett..

[6] Muñoz, J. A. Evolution of a continental collision belt: ECORS-Pyrenees crustal balanced cross-section. In *Thrust tectonics* 235–246 (Springer, 1992).

[7] Vergés, J., Fernàndez, M. & Martínez, A. The Pyrenean orogen: pre-, syn-, and post-collisional evolution. *Journal of the Virtual Explorer* 55–74 (2002).

[8] Mouthereau F (2014). Placing limits to shortening evolution in the Pyrenees: Role of margin architecture and implications for the Iberia/Europe convergence. Tectonics.

[9] Huyghe D, Mouthereau F, Castelltort S, Filleaudeau P-Y, Emmanuel L (2009). Paleogene propagation of the southern Pyrenean thrust wedge revealed by finite strain analysis in frontal thrust sheets: Implications for mountain building. Earth Planet. Sci. Lett..

[10] Fitzgerald PG, Muñoz JA, Coney PJ, Baldwin SL (1999). Asymmetric exhumation across the Pyrenean orogen: implications for the tectonic evolution of a collisional orogen. Earth Planet. Sci. Lett..

[11] Fillon C, van der Beek P (2012). Post-orogenic evolution of the southern P yrenees: constraints from inverse thermo-kinematic modelling of low-temperature thermochronology data. Basin Res..

[12] Huyghe D, Mouthereau F, Emmanuel L (2012). Oxygen isotopes of marine mollusc shells record Eocene elevation change in the Pyrenees. Earth Planet. Sci. Lett..

[13] Millán H (1995). Palaeo-elevation and effective elastic thickness evolution at mountain ranges: inferences from flexural modelling in the Eastern Pyrenees and Ebro Basin. Mar. Pet. Geol..

[14] Curry ME, van der Beek P, Huismans RS, Wolf SG, Muñoz J-A (2019). Evolving paleotopography and lithospheric flexure of the Pyrenean Orogen from 3D flexural modeling and basin analysis. Earth Planet. Sci. Lett..

[15] Séranne M (1999). The Gulf of Lion continental margin (NW Mediterranean) revisited by IBS: an overview. Geol. Soc. Lond. Spec. Publ..

[16] Suc J-P, Fauquette S (2012). The use of pollen floras as a tool to estimate palaeoaltitude of mountains: The eastern Pyrenees in the Late Neogene, a case study. Palaeogeogr. Palaeoclimatol. Palaeoecol..

[17] Wehr H, Chevrot S, Courrioux G, Guillen A (2018). A three-dimensional model of the Pyrenees and their foreland basins from geological and gravimetric data. Tectonophysics.

[18] Monod B, Regard V, Carcone J, Wyns R, Christophoul F (2016). Postorogenic planar palaeosurfaces of the central Pyrenees: Weathering and neotectonic records. Comptes Rendus Géoscience.

[19] Gunnell, Y. & Calvet, M. Comment on “Origin of the highly elevated Pyrenean peneplain” by Julien Babault, Jean Van Den Driessche, and Stéphane Bonnet, Sébastien Castelltort, and Alain Crave. *Tectonics* **25** (2006).

[20] Calvet M, Gunnell Y (2008). Planar landforms as markers of denudation chronology: an inversion of East Pyrenean tectonics based on landscape and sedimentary basin analysis. Geol. Soc. Lond. Spec. Publ..

[21] Babault J, Van den Driessche J, Bonnet S, Castelltort S, Crave A (2005). Origin of the highly elevated Pyrenean peneplain. Tectonics.

[22] Bosch GV (2016). Peneplanation and lithosphere dynamics in the Pyrenees. Comptes Rendus Géoscience.

[23] Campani M, Mulch A, Kempf O, Schlunegger F, Mancktelow N (2012). Miocene paleotopography of the Central Alps. Earth Planet. Sci. Lett..

[24] Mulch A (2016). Stable isotope paleoaltimetry and the evolution of landscapes and life. Earth Planet. Sci. Lett..

[25] Kohn MJ, Dettman DL (2007). Paleoaltimetry from stable isotope compositions of fossils. Rev. Mineral. Geochem..

[26] Royer A (2013). What does the oxygen isotope composition of rodent teeth record?. Earth Planet. Sci. Lett..

[27] Hays PD, Grossman EL (1991). Oxygen isotopes in meteoric calcite cements as indicators of continental paleoclimate. Geology.

[28] Zanchetta, G., Leone, G., Fallick, A. E. & Bonadonna, F. P. Oxygen isotope composition of living land snail shells: data from Italy. *Palaeogeography, Palaeoclimatology, Palaeoecology* 20–33 (2005).

[29] Quan C, Liu Y-S, Tang H, Utescher T (2015). Miocene shift of European atmospheric circulation from trade wind to westerlies. Sci. Rep..

[30] Huyghe D (2018). Impact of topography, climate and moisture sources on isotopic composition (δ18O & δD) of rivers in the Pyrenees: Implications for topographic reconstructions in small orogens. Earth Planet. Sci. Lett..

[31] Ortuño M (2013). Palaeoenvironments of the Late Miocene Prüedo Basin: implications for the uplift of the Central Pyrenees. J. Geol. Soc..

[32] Calvet M (2015). Cave levels as proxies for measuring post-orogenic uplift: Evidence from cosmogenic dating of alluvium-filled caves in the French Pyrenees. Geomorphology.

[33] Maurel O, Brunel M, Monié P (2002). Exhumation cénozoïque des massifs du Canigou et de Mont-Louis (Pyrénées orientales, France). Comptes Rendus Geosci..

[34] Baudelot S, Crouzel F (1974). La faune burdigalienne des gisements d’Espira-du-Conflent (Pyrénées-Orientales). Bull. Soc. D’Histoire Nat. Toulouse.

[35] Bache F (2010). Evolution of rifted continental margins: the case of the Gulf of Lions (Western Mediterranean Basin). Earth Planet. Sci. Lett..

[36] Jolivet L, Gorini C, Smit J, Leroy S (2015). Continental breakup and the dynamics of rifting in back-arc basins: The Gulf of Lion margin. Tectonics.

[37] McKenzie D (1978). Some remarks on the development of sedimentary basins. Earth Planet. Sci. Lett..

[38] Quirk DG, Rüpke LH (2018). Melt-induced buoyancy may explain the elevated rift-rapid sag paradox during breakup of continental plates. Sci. Rep..

[39] Gattacceca J (2007). Miocene rotation of Sardinia: New paleomagnetic and geochronological constraints and geodynamic implications. Earth Planet. Sci. Lett..

[40] Cabrera L, Roca E, Santanach P (1988). Basin formation at the end of a strike-slip fault: the Cerdanya Basin (eastern Pyrenees). J. Geol. Soc..

[41] Agustí J, Oms O, Furió M, Pérez-Vila M-J, Roca E (2006). The Messinian terrestrial record in the Pyrenees: the case of Can Vilella (Cerdanya Basin). Palaeogeogr. Palaeoclimatol. Palaeoecol..

[42] Clauzon G (2015). The Roussillon Basin (S. France): A case-study to distinguish local and regional events between 6 and 3 Ma. Mar. Pet. Geol..

[43] Saula, E. *et al*. Evolución geodinámica de la fosa del Empordà y las Sierras Transversales. *Acta Geologica Hispanica* 55–75 (1994).

[44] Cebriá Gómez, J. M., López Ruiz, J., Doblas, M. de las, Oyarzun, R. & Benito García, R. Geochemistry of the Quaternary alkali basalts of Garrotxa (NE Volcanic Province, Spain): A case of double enrichment of the mantle lithosphere (2000).

[45] Rowley DB (2007). Stable Isotope-Based Paleoaltimetry: Theory and Validation. Rev. Mineral. Geochem..

[46] Mulch A, Teyssier C, Cosca MA, Vanderhaeghe O, Vennemann TW (2004). Reconstructing paleoelevation in eroded orogens. Geology.

[47] Longinelli A (1984). Oxygen isotopes in mammal bone phosphate: a new tool for paleohydrological and paleoclimatological research?. Geochim. Cosmochim. Acta.

[48] Grimes ST, Hooker JJ, Collinson ME, Mattey DP (2005). Summer temperatures of late Eocene to early Oligocene freshwaters. Geology.

[49] Ehlers TA, Poulsen CJ (2009). Influence of Andean uplift on climate and paleoaltimetry estimates. Earth Planet. Sci. Lett..

[50] Aguilar, J.-P., Michaux, J. & Bachelet, B. Les nouvelles faunes de rongeurs proches de la limite Mio-Pliocène en Roussillon. *Palaeovertebrata* (1991).

[51] Huyghe D (2017). Significance of shallow-marine and non-marine algae stable isotope (δ18O) compositions over long periods: Example from the Palaeogene of the Paris Basin. Palaeogeogr. Palaeoclimatol. Palaeoecol..

[52] Pronin E, Pe\lechaty M, Apolinarska K, Pukacz A (2018). Oxygen stable isotope composition of carbonate encrustations of two modern, widely distributed, morphologically different charophyte species. Hydrobiologia.

[53] Joachimski MM (2009). Devonian climate and reef evolution: insights from oxygen isotopes in apatite. Earth Planet. Sci. Lett..

[54] Halas S, Skrzypek G, Meier-Augenstein W, Pelc A, Kemp HF (2011). Inter-laboratory calibration of new silver orthophosphate comparison materials for the stable oxygen isotope analysis of phosphates. Rapid Commun. Mass Spectrom..

