# Supplementary material for: Long-term dynamic topographic support during post-orogenic crustal thinning revealed by stable isotope (δ18O) paleo-altimetry in eastern Pyrenees
Source: Sci Rep. 2020 Feb 10;10:2267. doi: 10.1038/s41598-020-58903-w (PMC7010729; doi:10.1038/s41598-020-58903-w)
Supplement: Supplementary file 2 — Supplementary Dataset 2. [file 41598_2020_58903_MOESM2_ESM.docx]

**Supplementary data 2: information about the sampling sites and samples analyzed in the paper**

**Long-term dynamic topographic support during post-orogenic crustal thinning revealed by stable isotope (δ^18^O) paleo-altimetry in eastern Pyrenees**

**By Damien Huyghe, Frédéric Mouthereau, Loïc Ségalen and Marc Furio**

**Can Villella Section:**

The locality of Can Vilella encompasses up to nine different fossiliferous levels in about 25 meters of alluvial sediments, all of them containing small mammal teeth (Agustí et al., 2006; Furió and Agustí, 2017). The reference level is considered for this work is CV1. This is where all the samples studied in this work come from, because it is the richest and most representative level of the succession.

The recovery of the fossil material from Can Vilella 1 was carried in two steps. A first sample, which resulted in the discovery of the site, was taken during the project CAICYT n." PR83-3085-C02-02 in the middle 1980's (Agustí and Roca, 1987). A second and more meticulous sampling of this site was carried out in the early 2000's, including paleomagnetic and palynologic analyses, as a result of a broader coalescence of different projects (Agustí et al., 2006).

During the second phase in search of the fossil mammals and mollusks, hundreds of kilograms of sediments were water-screened using the Daams and Freudenthal (1988) techniques. The subsequent visual sorting processes under binocular microscopes resulted in the recovery of dozens of rodent, lagomorph and insectivore teeth (Agustí et al., 2006; Furió and Agustí, 2017), hundreds of carophyte oogonia and several freshwater gastropods.

This locality is confidently constrained within the stratigraphic succession of La Cerdanya Basin combining biostratigraphy and paleomagnetic techniques. For detailed images and descriptions of the fossil small mammal teeth found, the readers are referred to the abovementioned works of Agustí and Roca (1987), Agustí et al. (2006) and Furió and Agustí (2017).

**References**

Agustí, J. & Roca, E. 1987. Síntesis biostratigráfica de la Fosa de la Cerdanya (Pirineos Orientales). *Estudios Geológicos* 43, 521-529.

Agustí et al. 2006. The Messinian terrestrial record in the Pyrenees: The case of Can Vilella (Cerdanya Basin). *Palaeogeography, Palaeoclimatology, Palaeoecology* 238, 179-189.

Daams, R. & Freudenthal, M. 1988. Synopsis of the Dutch-Spanish collaboration program in the Aragonian type area, 1975-1986. *Scripta Geologica*, Special Issue 1, 3-18.

Furió, M. & Agustí, J. 2017. Latest Miocene insectivores from Eastern Spain: Evidence for enhanced latitudinal differences during the Messinian. *Geobios* 50, 123-140.

**Castellnou 3 cave:**

The Castelnou 3 Cave corresponds to a karstic cave. Rodent tooth were sampled by Jean-Pierre Aguilar during the 80’s. Information about the sampling site can be found in Aguilar et al., 1991.

**Reference**

Aguilar, J. P., Michaux, J., & Bachelet, B. (1991). Les nouvelles faunes de rongeurs proches de la limite Mio-Pliocène en Roussillon. Palaeovertebrata.

Photographs of the samples analyzed. The width of all pictures is 1.3 cm


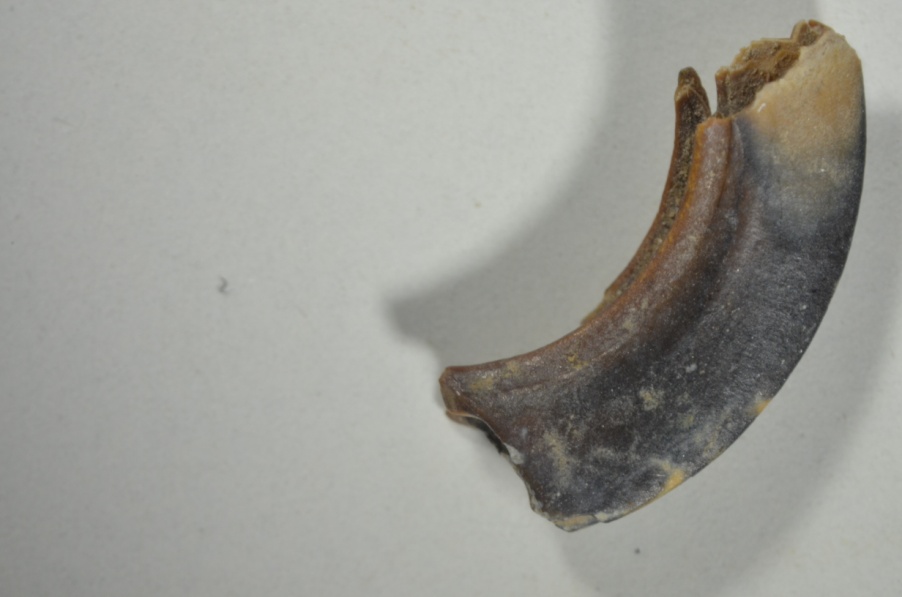


Lagormorph tooth (*Prolagus michauxi*), Can Villela section, Cerdanya Basin (Spain)


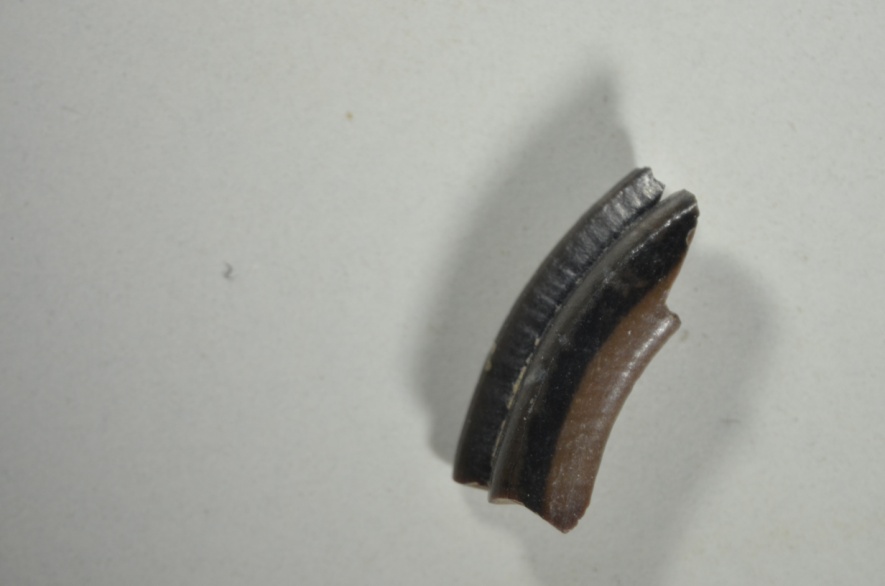


Lagormorph tooth, (*Prolagus michauxi*) Can Villela section, Cerdanya Basin (Spain)


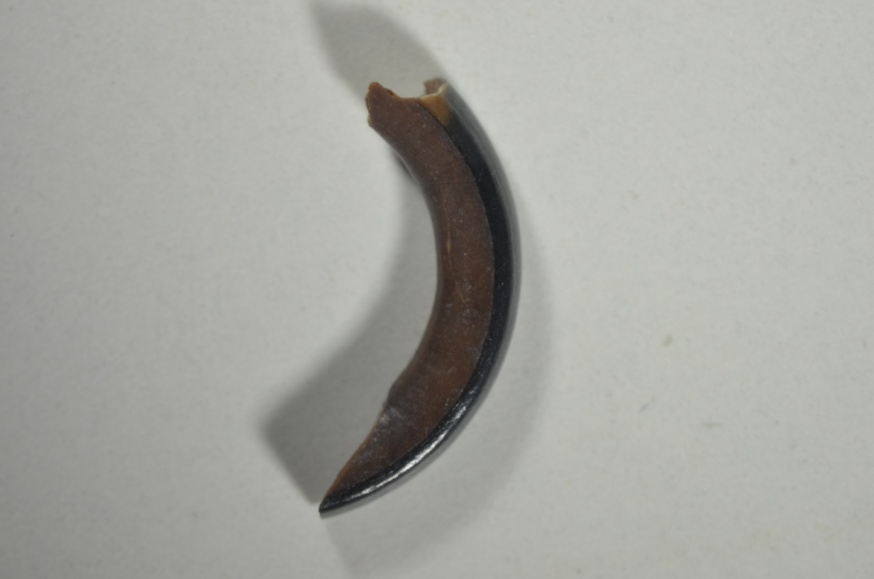


Undetermined rodent incisor, Can Villela section, Cerdanya Basin (Spain)


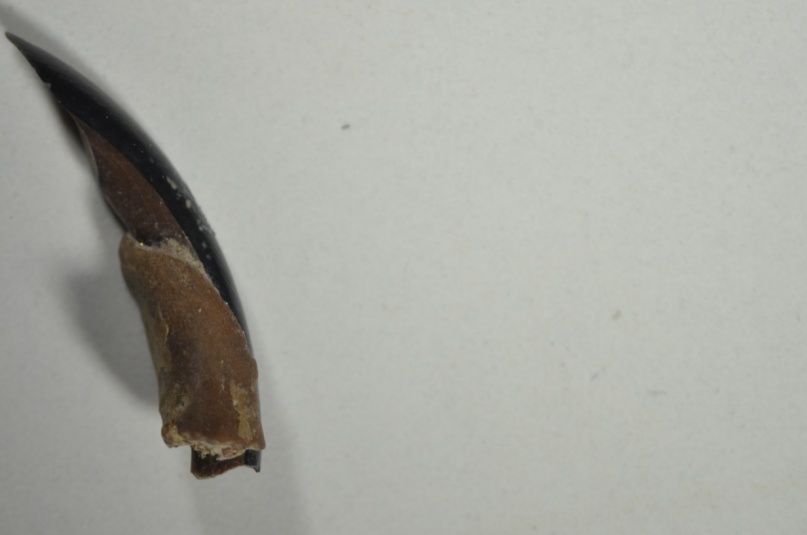


Undetermined rodent incisor, Can Villela section, Cerdanya Basin (Spain)


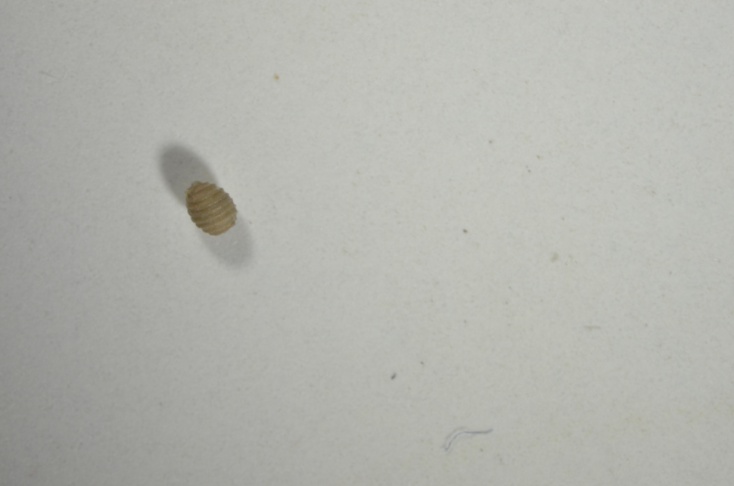


Charophyte oogonia (*Lychnothamnus barbatus*), Can Villela section, Cerdanya Basin (Spain)


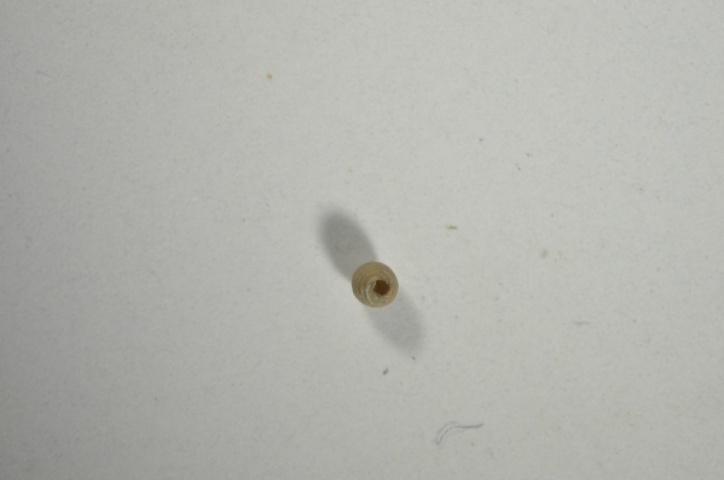


Charophyte oogonia (*Lychnothamnus barbatus*), Can Villela section, Cerdanya Basin (Spain)


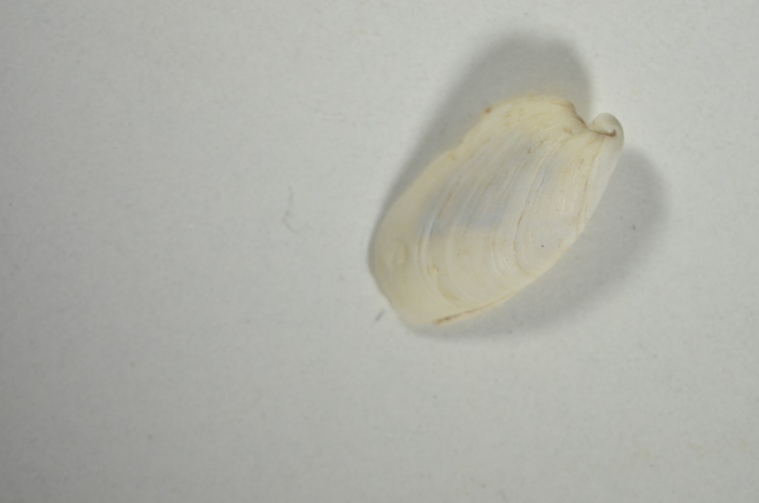


*Testacella,* Can Villela section, Cerdanya Basin (Spain)


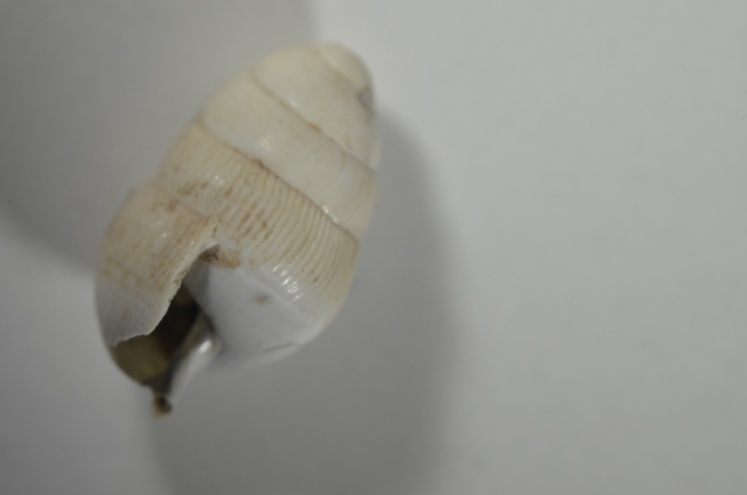


Clausilid shells*,* Can Villela section, Cerdanya Basin (Spain)


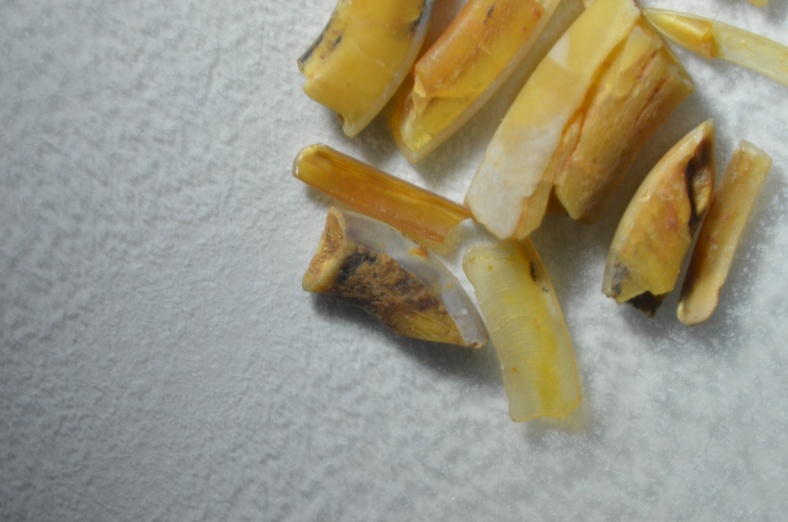


Undetermined rodent incisor, Castellnou 3 Cave, Roussillon Basin (France)
